# The human anti-CD40 agonist antibody mitazalimab (ADC-1013; JNJ-64457107) activates antigen-presenting cells, improves expansion of antigen-specific T cells, and enhances anti-tumor efficacy of a model cancer vaccine in vivo

**DOI:** 10.1007/s00262-021-02932-5

**Published:** 2021-05-05

**Authors:** Adnan Deronic, Anneli Nilsson, Mia Thagesson, Doreen Werchau, Karin Enell Smith, Peter Ellmark

**Affiliations:** 1 Alligator Bioscience AB, Medicon Village, 223 81 Lund, Sweden; 2 Department of Immunotechnology, Lund University, Lund, Sweden

**Keywords:** Cancer immunotherapy, CD40 agonist antibody, Dendritic cell activation, Cancer vaccine

## Abstract

Non-responders to checkpoint inhibitors generally have low tumor T cell infiltration and could benefit from immunotherapy that activates dendritic cells, with priming of tumor-reactive T cells as a result. Such therapies may be augmented by providing tumor antigen in the form of cancer vaccines. Our aim was to study the effects of mitazalimab (ADC-1013; JNJ-64457107), a human anti-CD40 agonist IgG1 antibody, on activation of antigen-presenting cells, and how this influences the priming and anti-tumor potential of antigen-specific T cells, in mice transgenic for human CD40. Mitazalimab activated splenic CD11c^+^ MHCII^+^ dendritic cells and CD19^+^ MHCII^+^ B cells within 6 h, with a return to baseline within 1 week. This was associated with a dose-dependent release of proinflammatory cytokines in the blood, including IP-10, MIP-1α and TNF-α. Mitazalimab administered at different dose regimens with ovalbumin protein showed that repeated dosing expanded ovalbumin peptide (SIINFEKL)-specific CD8^+^ T cells and increased the frequency of activated ICOS^+^ T cells and CD44^hi^ CD62L^−^ effector memory T cells in the spleen. Mitazalimab prolonged survival of mice bearing MB49 bladder carcinoma tumors and increased the frequency of activated granzyme B^+^ CD8^+^ T cells in the tumor. In the ovalbumin-transfected tumor E.G7-OVA lymphoma, mitazalimab administered with either ovalbumin protein or SIINFEKL peptide prolonged the survival of E.G7-OVA tumor-bearing mice, as prophylactic and therapeutic treatment. Thus, mitazalimab activates antigen-presenting cells, which improves expansion and activation of antigen-specific T cells and enhances the anti-tumor efficacy of a model cancer vaccine.

**Supplementary Information:**

The online version contains supplementary material available at 10.1007/s00262-021-02932-5.

## Introduction

Over the past decade, cancer immunotherapy has seen major advancements driven largely by encouraging results achieved with checkpoint inhibitors (CPI). However, a large group of patients remain non-responsive to CPI, an observation which has been linked to the state of the immune landscape within the tumor. Generally, tumors with a high degree of T cell infiltration are more responsive to CPI than tumors with a low degree of T cell infiltration [1–3]. Factors that contribute to a low tumor T cell infiltration are lack of tumor neoantigens, or low tumor mutational burden, absence of antigen-presenting cells (APC), poor T cell priming and impaired T cell trafficking to the tumor tissue [4, 5]. Thus, immunotherapy aiming to improve APC function, e.g., CD40 agonist antibodies, has the potential to provide clinical benefit in tumors that lack tumor-reactive T cell infiltrate due to insufficient APC activation and T cell priming. The effect of CD40 agonist antibodies can be further enhanced with radiotherapy, chemotherapy, or cancer vaccines, which are viable approaches for increasing availability of tumor antigens [6, 7].

CD40 is a cell-surface glycoprotein that belongs to the tumor necrosis factor receptor superfamily and is expressed on APC such as dendritic cells (DC), B cells and macrophages, as well as epithelial and endothelial cells and certain types of tumor cells [8]. By interactions with CD40 ligand, mainly expressed by T cells and platelets, CD40 signaling mediates activation of DC resulting in upregulation of MHC molecules, co-stimulatory markers (e.g., CD80 and CD86) and production of proinflammatory cytokines (e.g., IL-6, IL-12 and TNF-α) [9, 10]. This improves the ability of DC to prime naïve CD4^+^ and CD8^+^ T cells. Further, activation of DC via CD40 stimulates efficient cross-presentation, which is crucial for inducing a CD8^+^ T cell response towards tumor antigen, both in situ tumor antigens and therapeutic vaccines [11–13]. There is thus a strong rationale for combining CD40 agonist antibodies with vaccines. Recent preclinical data demonstrating that CD40 agonist therapy significantly enhances vaccination therapy combined with PD-1 blockade, further support this approach [14].

Several CD40 agonist antibodies are currently evaluated in clinical trials for treatment of cancer [7, 15]. Some of these are assessed in combination with CPI, primarily anti-PD-(L)1 antibodies, but also with chemotherapy or cancer vaccine [16, 17]. Mitazalimab (ADC-1013; JNJ-64457107), a human anti-CD40 agonist IgG1 antibody, has been evaluated in two phase 1 clinical studies in patients with advanced stage solid tumors. In these studies, mitazalimab was shown to be well-tolerated at high dose levels and induced CD40-mediated agonistic effects such as APC activation and cytokine release [18, 19]. However, the kinetics of APC activation mediated by human CD40 agonists have not been explored in detail, or how different dose regimens of CD40 agonists affect priming of T cells and the subsequent T cell activity.

Here, we use preclinical in vivo models to demonstrate that mitazalimab activates APC such as DC and that repeated treatment with mitazalimab improves expansion and activation of antigen-specific T cells. Finally, we show that mitazalimab enhances the anti-tumor efficacy of a model cancer vaccine.

## Materials and methods

### Cell lines

The murine bladder carcinoma cell line MB49 was purchased from EMD Millipore Corporation and the murine T lymphoma cell lines EL-4 and E.G7-OVA were both purchased from ATCC. MB49 and EL-4 cells were cultured in DMEM supplemented with 10% fetal bovine serum at 37°C and 5% CO_2_. E.G7-OVA cells were cultured in RPMI-1640 supplemented with 10% fetal bovine serum, 10 mM HEPES, 2 mM sodium pyruvate, 50 μM β-mercaptoethanol, and 0.4 mg/ml G418 at 37°C and 5% CO_2_.

### Mice and ethical considerations

All in vivo experiments were performed in C57Bl/6 female homozygous human CD40 transgenic (hCD40tg) mice, between 9 and 14 weeks of age. The hCD40tg mice express the human CD40 gene under control of the murine CD40 promoter and regulatory elements on a murine CD40 negative background. The hCD40tg mice show expression of human CD40 comparable to murine CD40 in wildtype mice on DC, but lower expression on B cells [20]. Animals were housed according to regional regulations. All animal experiments were approved by the regional Ethics Committee in Lund/Malmö, Sweden.

### In vivo experimental design

APC activation kinetics were studied in naïve mice administered a single dose of 100 µg mitazalimab or control IgG (MabThera; Roche) intraperitoneally (i.p.). Mice were sacrificed at 6 h, 24 h, 48 h, 72 h, 96 h, or 7 days after treatment and spleens collected for flow cytometry.

APC responses and expansion of ovalbumin (OVA)-specific T cells were studied in the OVA vaccination model. Mice were immunized with 200 µg OVA protein (Sigma-Aldrich) intravenously (i.v.) on three occasions, 7 days between each occasion. Additionally, the mice were administered 100 µg mitazalimab i.p. either every 2–3 days, every 7 days or every 14 days starting from the first OVA injection. A cohort of mice was sacrificed from each group every 7 days for 6 weeks and spleens and blood, collected via vena cava in lithium heparin S-Monovette tubes (Sarstedt), were used for flow cytometry.

For prophylactic vaccination studies in the E.G7-OVA tumor model, mice were immunized with 200 µg OVA protein i.v. on two occasions, 7 days between, and were also administered 100 µg mitazalimab i.p. either every 2–3 days or every 7 days, starting from the first OVA injection. Control IgG given at a dose of 100 µg every 2–3 days was used for comparison. On day 14 after the first OVA injection, mice were either sacrificed and spleens collected for flow cytometry, or mice were inoculated with 1 × 10^6^ E.G7-OVA cells subcutaneously (s.c.) on the right flank. Tumor growth was continuously measured and survival of the mice was assessed. Mice were taken out of the experiment upon signs of ill health, wounding of tumors, or once tumors approached a volume of 2 cm^3^. Complete responder mice were rechallenged with 1 × 10^6^ E.G7-OVA cells, inoculated s.c. on the left flank. These mice were also inoculated with an additional tumor, 1 × 10^6^ EL-4 cells s.c. on the right flank.

For therapeutic vaccination studies, mice were first inoculated with 1 × 10^6^ E.G7-OVA cells s.c. on the right flank. Following inoculation, 100 µg mitazalimab and/or 10 µg OVA peptide (SIINFEKL, GenScript) were administered s.c. on the left flank, either as single agent therapy or in the same formulation, on the same day and once more 7 days later. Alternatively, the first treatment was administered on day 3 post-inoculation and the second on day 10.

Anti-tumor efficacy studies were performed in the MB49 tumor model with mitazalimab monotherapy. Mice were inoculated with 0.25 × 10^6^ MB49 cells s.c. on the right flank. On day 7, 10, and 13 post-inoculation, 100 µg mitazalimab or control IgG was administered either i.p. or peritumorally (p.t.).

Alternatively, 10 µg, 30 µg, 100 µg or 300 µg mitazalimab was administered i.p. to MB49-tumor bearing mice on day 7, 10 and 13 post-inoculation. Blood was collected via vena saphena in Microvette CB 300 Z tubes (Sarstedt) 24 h after the first dose and serum used for analysis by Meso Scale Diagnostics (MSD). Mice were sacrificed 24 h after the final dose and spleens and blood, collected via vena cava in lithium heparin S-Monovette tubes, were used for flow cytometry.

### Antibodies and flow cytometry

Dissected spleens were mashed in 70-µm cell strainers. Blood samples were treated with BD Pharm Lyse (BD Biosciences) according to manufacturer instructions. Tumors were cut into small pieces and enzymatically digested with liberase TL (Roche) and DNase I (Roche) at 37°C according to manufacturer instructions, and subsequently mashed in 70-µm cell strainers. The single-cell suspensions were Fc blocked (clone 2.4G2; BD Biosciences).

The following anti-mouse antibodies used were purchased from BioLegend: anti-CD11c (clone N418) and anti-CD19 (clone 6D5). The following anti-mouse antibodies used were purchased from Thermo Fisher Scientific: anti-granzyme B (clone NGZB) and anti-I-A/I-E (clone M5/114.15.2). The following anti-mouse antibodies were purchased from BD Biosciences: anti-B220 (clone RA3-6B2), anti-CD3 (clone 145-2C11), anti-CD4 (clone RM4-5), anti-CD8 (clone 53-6.7), anti-CD11b (clone M1/70), anti-CD11c (clone HL3), anti-CD44 (clone IM7), anti-CD45 (clone 30-F11), anti-CD62L (clone MEL-14), anti-CD80 (clone 16-10A1), anti-CD86 (clone GL1), anti-ICOS (clone 7E.17G9), anti-I-A/I-E (clone 2G9) and anti-NK1.1 (clone PK136). Anti-CD8 (clone KT15) and the iTag Tetramer—H-2 Kb OVA (SIINFEKL) were both purchased from MBL International. The fixable viability stain FVS450 or FVS780 (BD Biosciences) were used to stain non-viable cells. The samples were analyzed using FACSVerse (BD Biosciences), and data were processed using FlowJo software. Gates for the presented cell populations were defined by use of fluorescence minus one (FMO) controls.

### MSD analysis

Blood samples were centrifuged at 2000 × *g* for 10 min and the resulting serum layer stored at −80°C until further use. The samples were thawed, diluted fivefold or sixfold, and analyzed with the V-PLEX Mouse Cytokine 19-Plex Kit (MSD) according to manufacturer instructions.

### Statistical analyses

Survival data were plotted by the Kaplan–Meier method and statistical significance analyzed by the log-rank test. Where indicated, the difference between groups was evaluated using Mann–Whitney *U* test or ordinary two-way ANOVA and Šídák’s multiple comparisons test. *P* values less than 0.05 were considered significant. Asterisks indicate the confidence intervals (*, *P* ≤ 0.05; **, *P* ≤ 0.01; ***, *P* ≤ 0.001; ****, *P* ≤ 0.0001). All statistical analyses were performed using GraphPad Prism software.

## Results

### Mitazalimab rapidly and transiently activates DC and B cells

**Fig. 1:**
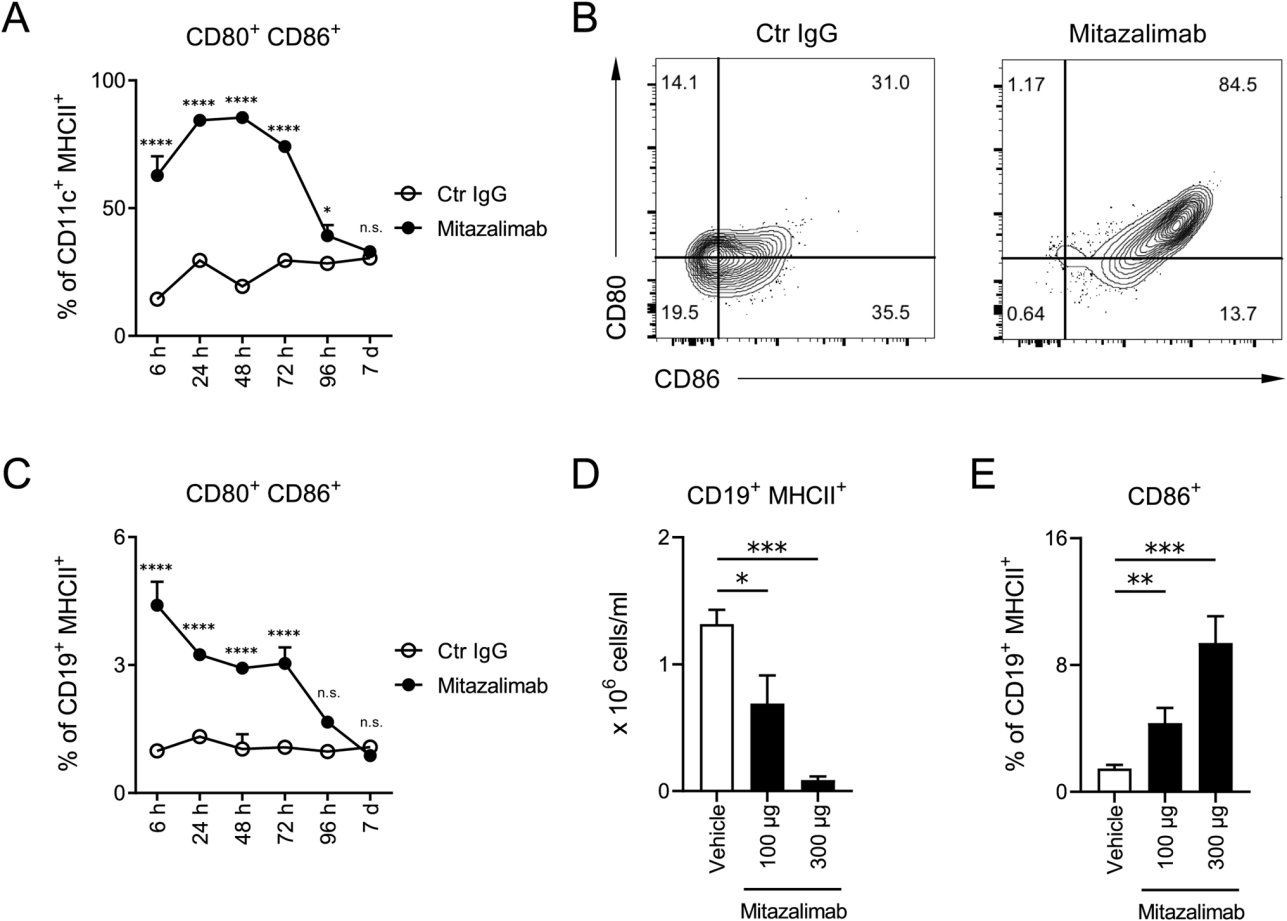
Mitazalimab activates splenic DC and B cells—Naïve hCD40tg mice were administered a single dose of 100 µg mitazalimab or Ctr IgG i.p. and spleens collected for flow cytometry from 6 h up to 7 days after treatment. **a** Frequency of activated CD80^+^ CD86^+^ splenic DC (CD11c^+^ MHCII^+^), **b** representative FACS plots from the 24 h time point in **a**, **c** frequency of activated CD80^+^ CD86^+^ splenic B cells (CD19^+^ MHCII^+^). MB49 tumor-bearing mice were administered repeated dosing of 100 or 300 µg mitazalimab i.p. on day 7, 10 and 13 post-inoculation, and 24 h after the final dose, the mice were anaesthetized and blood collected via vena cava for flow cytometry, **d** number of circulating B cells per ml of blood, **e** frequency of activated CD86^+^ circulating B cells. *n* = 3 per group in **a**–**c**; *n* = 8 per group in **d**–**e**. Statistical significance was analyzed by ordinary two-way ANOVA and Šídák’s multiple comparisons test in **a** and **c**, and by Mann–Whitney *U* test in **d** and **e**. Error bars for all data points are included, although not always visible, and indicate ± SEM

The primary mode of action of mitazalimab is activation of APC via CD40. APC activation has been demonstrated by in vitro culture of human monocyte-derived DC, which upregulated expression of CD80 and CD86 following exposure to mitazalimab [20]. To gain a better understanding of the kinetics of APC activation following systemic administration of mitazalimab in vivo, naïve hCD40tg mice were given a single dose of mitazalimab and the activation of DC and B cells monitored by flow cytometry analyses of the spleen. Within 6 h, mitazalimab induced a potent increase of both CD80 and CD86 on CD11c^+^ MHCII^+^ DC, compared to mice which had received control IgG (Fig. 1a, b). The maximal effect was reached at approximately 48 h post-treatment, and after this point, the levels of CD80 and CD86 started to decrease and returned to baseline by day 7 post-treatment. PD-L1 was also increased on DC with similar kinetics (data not shown). A similar effect on CD80 and CD86 was observed for CD19^+^ MHCII^+^ B cells; however, the activation of these cells peaked already within 6 h post-treatment (Fig. 1c). The frequency of activated B cells following mitazalimab treatment was clearly lower compared to the DC. This was an expected observation as only a fraction of the circulating B cells express hCD40 in the hCD40tg mouse model used [20].

**Fig. 2:**
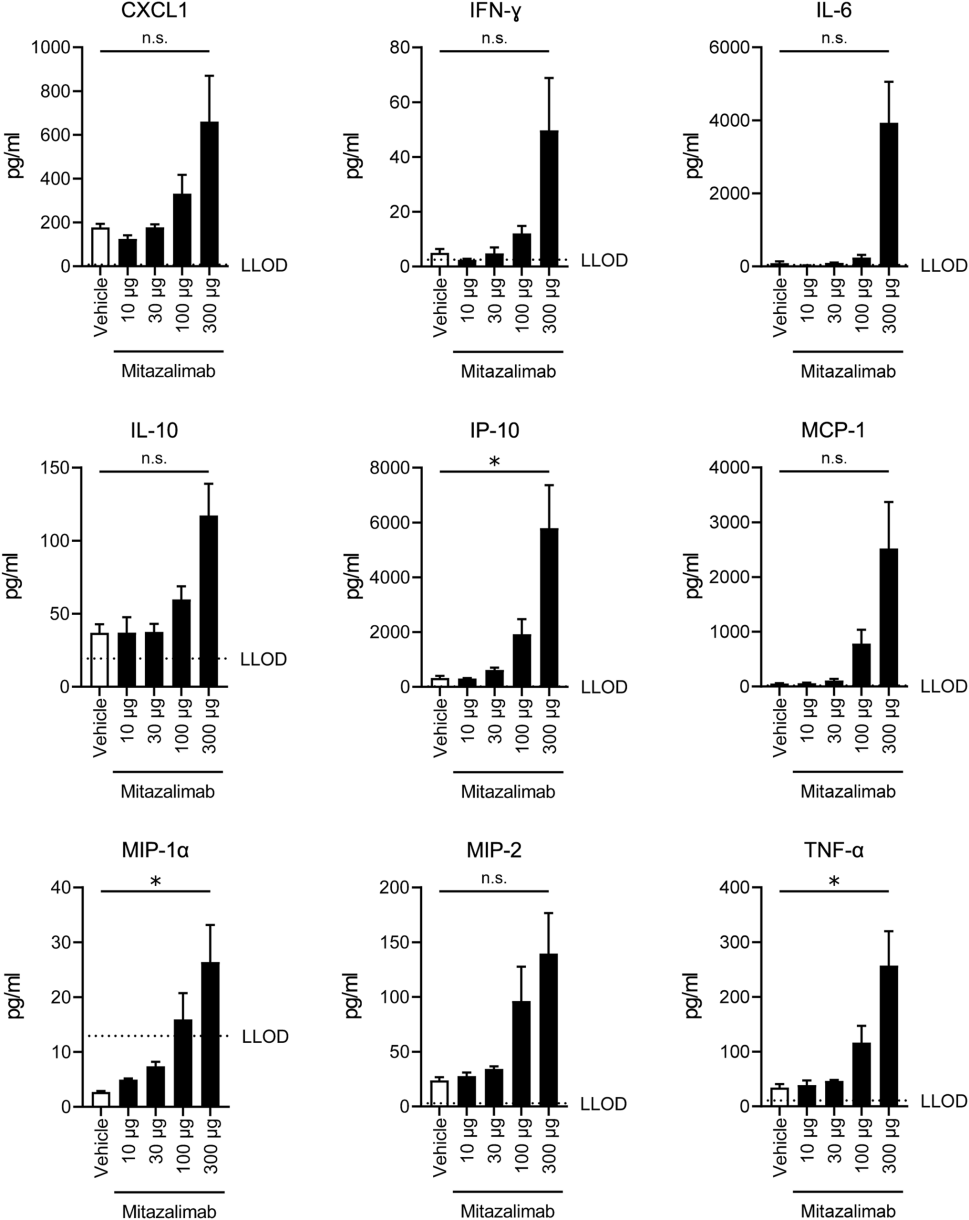
Mitazalimab induces the release of proinflammatory cytokines and chemokines in the blood**—**MB49 tumor-bearing hCD40tg mice were administered a single dose of 10, 30, 100 or 300 µg mitazalimab i.p. on day 7 post-inoculation, and 24 h after dosing, blood was collected via vena saphena for MSD analysis. The V-PLEX Mouse Cytokine 19-Plex Kit was used to measure CXCL1 (KC/GRO), IFN-γ, IL-1β, IL-2, IL-4, IL-5, IL-6, IL-9, IL-10, IL-12p70, IL-15, IL-17A/F, IL-27p28/IL-30, IL-33, IP-10, MCP-1, MIP-1α, MIP-2 and TNF-α. Only the cytokines/chemokines that showed a consistent change across two experiments are shown. *n* = 2–5 per group. Statistical significance was analyzed by Mann–Whitney *U* test. The lower limit of detection (LLOD) is defined by a horizontal dotted line. All error bars indicate ± SEM

As mitazalimab is intended for treatment of cancer, pharmacodynamic markers were also assessed in tumor-bearing hCD40tg mice. Repeated exposure to mitazalimab resulted in reduced numbers of circulating B cells (Fig. 1d), and upregulation of CD86 on remaining B cells (Fig. 1e), which correlates with observations in blood samples obtained from cancer patients that had undergone treatment with mitazalimab [18, 19], as well as other anti-CD40 antibodies evaluated in a clinical setting [21–24]. Additionally, mitazalimab administered once, at dose levels ranging from 10 to 300 µg resulted in a dose-dependent significant increase of IP-10, MIP-1α and TNF-α, and non-significant increase of CXCL1, IFN-γ, IL-6, IL-10, MCP-1 and MIP-2 (Fig. 2). Similar effects on cytokine release were observed in non-tumor-bearing mice (data not shown). These observations further highlight the immune-activating capacity of mitazalimab.

### Mitazalimab results in expansion of antigen-specific T cells in a vaccination model

**Fig. 3:**
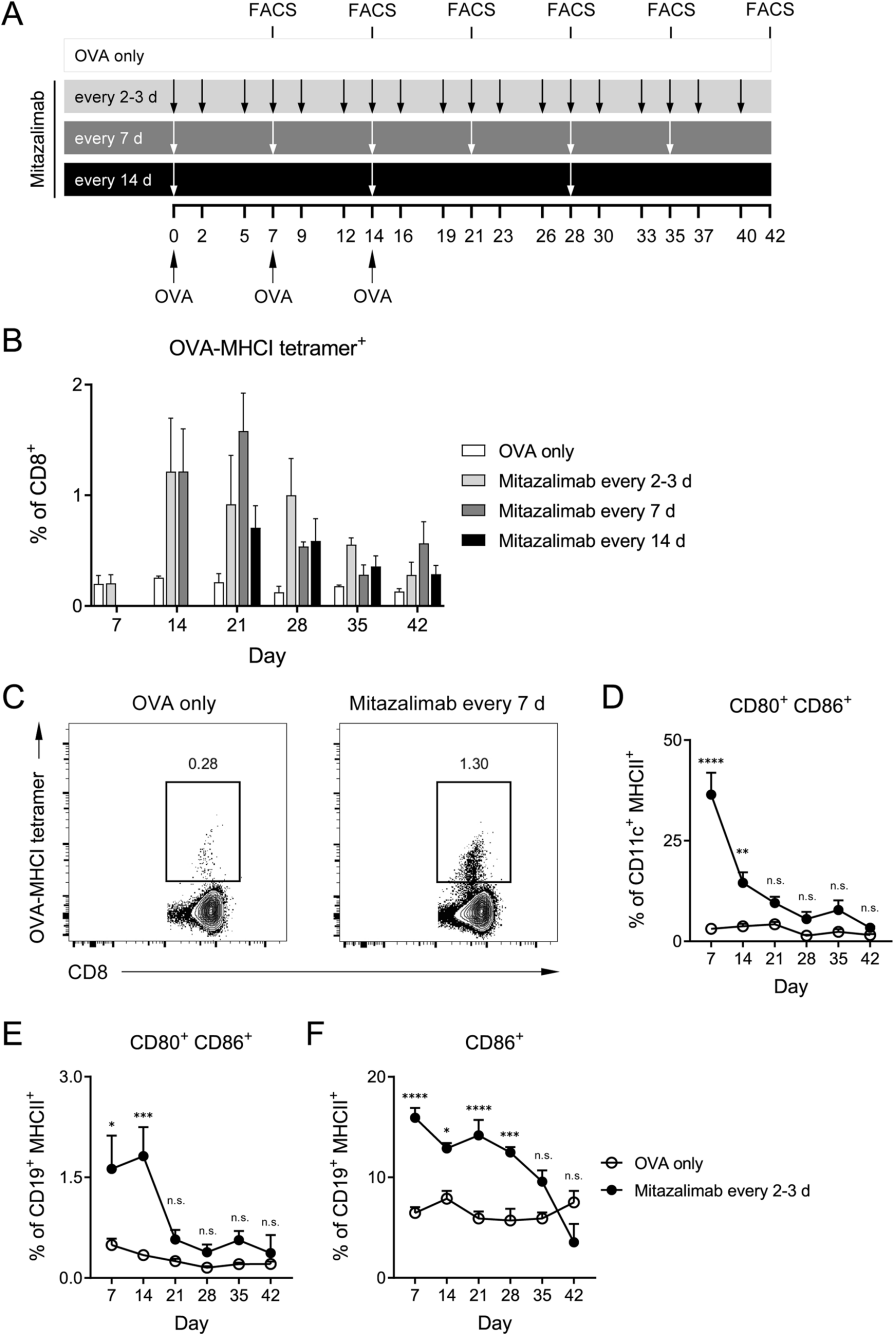
Repeated dosing of mitazalimab in OVA-rechallenged mice results in expansion of OVA-specific CD8^+^ T cells—Naïve hCD40tg mice were immunized with 200 µg OVA protein i.v. on 3 occasions, 7 days between. The mice were divided into different cohorts, wherein each cohort was given 100 µg mitazalimab i.p. at different dose regimens. One cohort received no mitazalimab treatment (OVA only). A subset of mice from each cohort was sacrificed once weekly for 6 weeks after the first immunization and spleens and blood collected for flow cytometry. **a** Overview of the experimental set-up, **b** frequency of OVA-specific (SIINFEKL-MHCI tetramer^+^) splenic CD8^+^ T cells, **c** representative FACS plots from two groups from the day 14 time point in **b**, **d** frequency of activated CD80^+^ CD86^+^ splenic DC (CD11c^+^ MHCII^+^), **e** frequency of activated CD80^+^ CD86^+^ splenic B cells (CD19^+^ MHCII^+^), **f** frequency of activated CD86^+^ circulating B cells. *n* = 3–4 per group in **a**–**f**. Statistical significance was analyzed by ordinary two-way ANOVA and Šídák’s multiple comparisons test in **b**, **d**–**f**. No relevant comparisons were significant in **b**. All error bars indicate ± SEM

To explore how the effect of mitazalimab on DC activation would impact priming of T cells, an OVA vaccination model was used. Briefly, hCD40tg mice were immunized with OVA protein on three occasions, and different dose regimens of mitazalimab were given according to Fig. 3a. A cohort of mice from each group was sacrificed once weekly after the first OVA immunization and flow cytometry analyses were performed on spleens to assess the frequency of OVA-specific T cells using OVA peptide (SIINFEKL)-MHCI tetramers. Seven days after the first OVA immunization, mitazalimab in combination with a single dose of OVA did not result in a detectable expansion of OVA-specific CD8^+^ T cells, compared to mice which received OVA only. However, when mitazalimab was administered in combination with repeated OVA immunizations, there appeared to be an expansion of these cells from day 14 and onwards, although these effects were not significant (Fig. 3b, c). This observation was most pronounced in mice which received mitazalimab every 2–3 days or every 7 days. Thus, repeated treatment with mitazalimab appeared to improve expansion of OVA-specific T cells in mice rechallenged with OVA.

Over time, the continuous and frequent exposure to mitazalimab resulted in a more rapid downregulation of CD80 and CD86 on DC and B cells. This effect was observed when mitazalimab was administered every 2–3 days, as this dose regimen resulted in a clearly reduced effect of mitazalimab on activation of both DC (Fig. 3d) and B cells (Fig. 3e) in the spleen. A similar trend was observed for circulating B cells, where the effect of mitazalimab on levels of CD86 also gradually decreased, although at a slower rate (Fig. 3f). This also correlated with a reduction of an initially increased frequency of DC in the spleen (Suppl. Figure 1), and reduced levels of proinflammatory cytokines (e.g., IFN-γ and IL-6) in the blood after the first week of treatment (data not shown). These observations suggest that continuous and frequent exposure to mitazalimab may result in immune exhaustion; however, it cannot be excluded that development of neutralizing anti-mitazalimab antibodies may affect the readout in this setting.

### Prophylactic treatment with mitazalimab and a model cancer vaccine delays tumor growth and prolongs survival

**Fig. 4:**
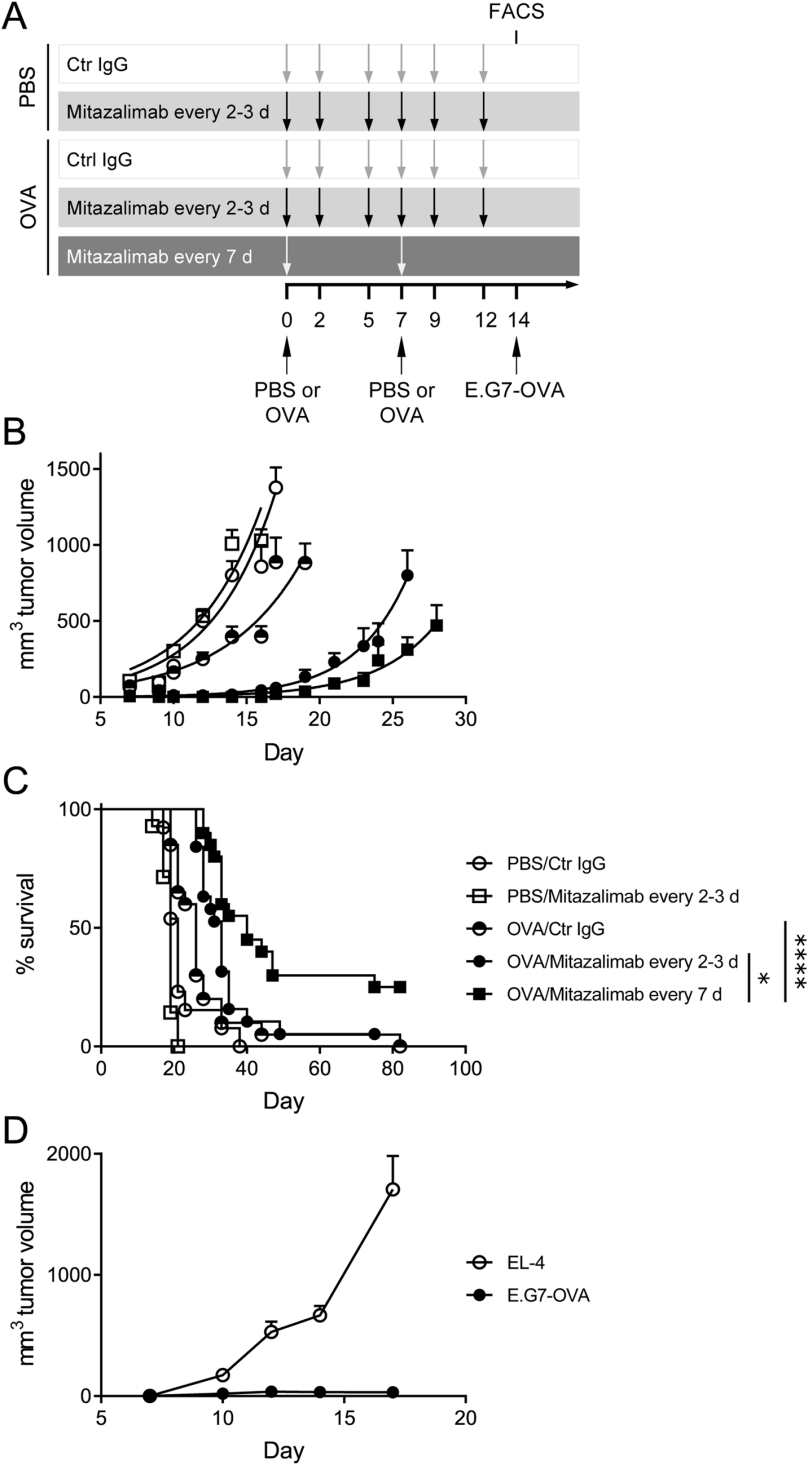
Prophylactic treatment with mitazalimab and OVA protein prolongs the survival of E.G7-OVA tumor-bearing mice and induces immunological memory against OVA—Naïve hCD40tg mice were immunized twice with 200 µg OVA protein or PBS control i.v., 7 days between. Mitazalimab or Ctr IgG (100 µg) were given i.p. either every 2–3 days or every 7 days during a 12-day period, starting from the first OVA injection. On day 14, 1 × 10^6^ E.G7-OVA cells were inoculated s.c. **a** Overview of the experimental setup, **b** E.G7-OVA tumor growth throughout the study. Tumor growth is displayed until the point where the first mouse in each group approaches the ethical tumor volume limit of 2 cm^3^ and is sacrificed, **c** frequency of survival throughout the study. Pooled data from two identical experiments are shown in **b** and **c**. The complete responders in **c** were again inoculated with 1 × 10^6^ E.G7-OVA cells s.c. on one flank and 1 × 10^6^ EL-4 cells on the other flank, **d** E.G7-OVA and EL-4 tumor growth of the complete responders. Tumor growth is displayed until the point where the first complete responder is sacrificed. *n* = 8–10 per group, per experiment, in **a**–**c**; *n* = 5 in total in **d**. Statistical significance was analyzed by the log-rank test in **c**. All error bars indicate ± SEM

As mitazalimab treatment induced expansion of OVA-specific T cells in the OVA vaccination model, this model was subsequently utilized to evaluate the potential for mitazalimab to improve the anti-tumor efficacy of OVA, acting as a model cancer vaccine. Thus, mice were immunized with OVA twice, and mitazalimab was given either every 2–3 days or every 7 days (Fig. 4a), as these dose regimens appeared to result in expansion of OVA-specific T cells (Fig. 3b). On day 14, E.G7-OVA (EL-4 lymphoma transfected with OVA) tumors were inoculated and tumor growth and survival of the mice was monitored. No additional treatments were given following tumor inoculation. The results show that, although immunization with OVA only resulted in slightly delayed tumor growth, this effect was significantly improved when OVA immunization was combined with mitazalimab (Fig. 4b). Interestingly, the dose regimen where mitazalimab was given every 7 days induced the most potent tumor growth delay and significantly prolonged the overall survival (Fig. 4c).

To demonstrate the immunological memory to OVA, complete responder mice which had all received OVA and mitazalimab, were inoculated again with E.G7-OVA cells on one flank and the parental EL-4 cells, which lack OVA expression, on the other. In these mice, E.G7-OVA tumors displayed no tumor growth, while the EL-4 tumors grew rapidly (Fig. 4d). This observation would suggest that repeated administration of OVA strongly skews the immune response to this particular model antigen.

**Fig. 5:**
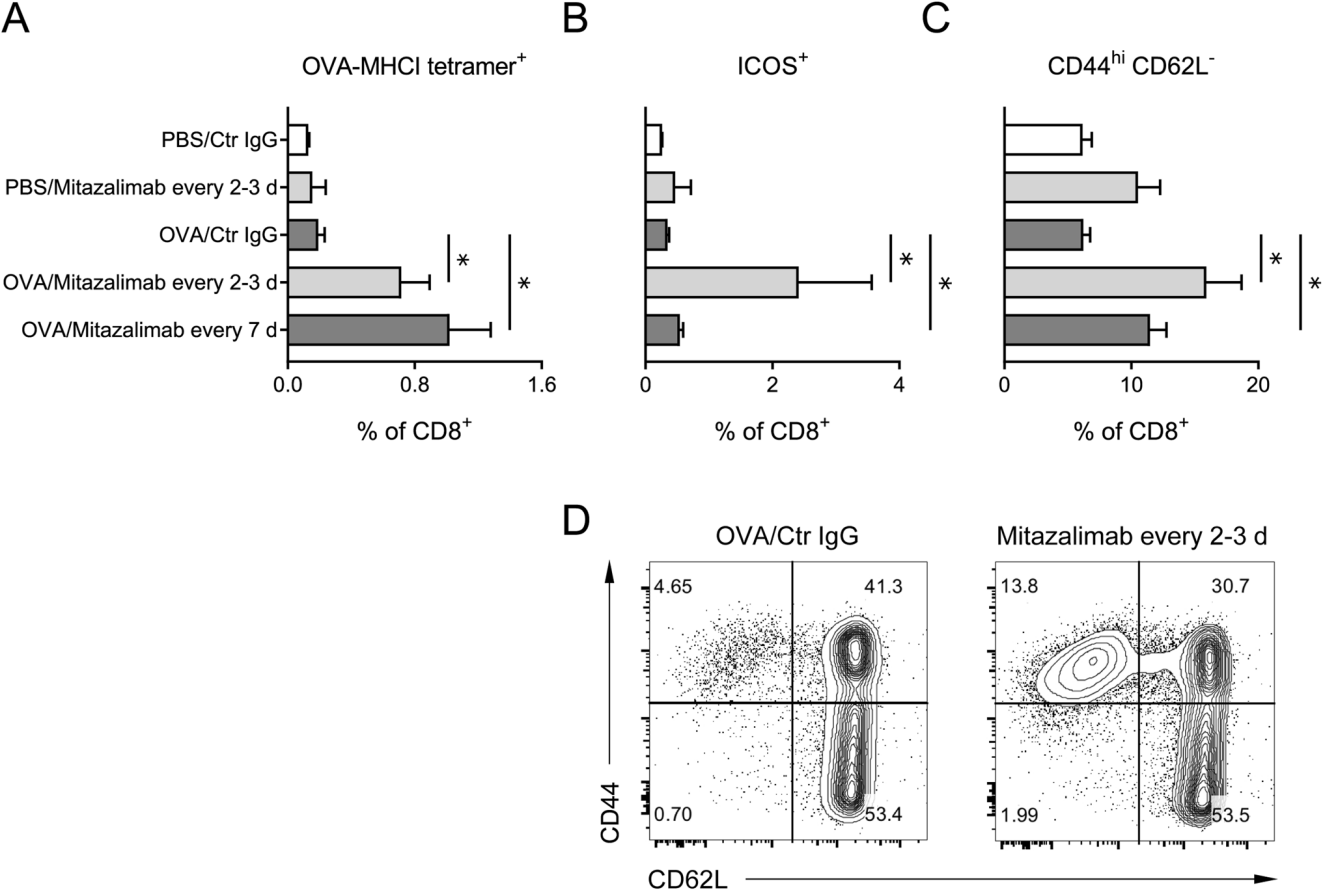
Mitazalimab treatment results in improved T cell activation—A cohort of mice from each treatment group in Fig. 4a was sacrificed before tumor inoculation on day 14 and spleens collected for flow cytometry. **a** Frequency of OVA-specific (SIINFEKL-MHCI tetramer^+^) splenic CD8^+^ T cells, **b** frequency of ICOS^+^ splenic CD8^+^ T cells, **c** frequency of CD44^hi^ CD62L^−^ splenic CD8^+^ T cells, **d** representative FACS plots from two of the treatment groups in **c**. *n* = 3–4 per group in **a**–**d**. Statistical significance was analyzed by Mann–Whitney *U* test. All error bars indicate ± SEM

To gain a better understanding of the effect of mitazalimab on T cell activation in this setting, a cohort of mice from each group was sacrificed before tumor inoculation and spleens collected for flow cytometry analyses of various T cell activation markers. Here, OVA immunization in combination with mitazalimab resulted in a significant expansion of OVA-specific CD8^+^ T cells for both mitazalimab dose regimens, compared to mice receiving OVA and control IgG (Fig. 5a). In addition, the T cell activation marker ICOS was noticeably upregulated on both CD8^+^ (Fig. 5b) and CD4^+^ T cells (Suppl. Figure 2a) in mice treated with mitazalimab every 2–3 days. At the time point of analysis, mice which received mitazalimab every 7 days had not been exposed to mitazalimab for 7 days, and thus it is likely that the levels of ICOS had almost returned to baseline, which would explain the discrepancy in ICOS levels between the two mitazalimab dose regimen groups. Mitazalimab treatment also resulted in increased frequency of CD44^hi^ CD62L^−^ effector memory CD8^+^ (Fig. 5c, d) and CD4^+^ T cells (Suppl. Figure 2b). No major effects of mitazalimab were observed for total splenic CD4^+^ (Suppl. Figure 2c) and CD8^+^ T cells (Suppl. Figure 2d), although mitazalimab given every 2–3 days increased the CD4^+^/CD8^+^ T cell ratio in mice immunized with OVA.

Collectively, these data demonstrate that prophylactic treatment with mitazalimab and OVA results in improved T cell activation and expansion of OVA-specific CD8^+^ T cells, which have the capacity to eradicate OVA-expressing tumors. This generates an immunological memory specifically for tumors expressing this antigen.

### Therapeutic treatment with mitazalimab and a model cancer vaccine delays tumor growth and prolongs survival

**Fig. 6:**
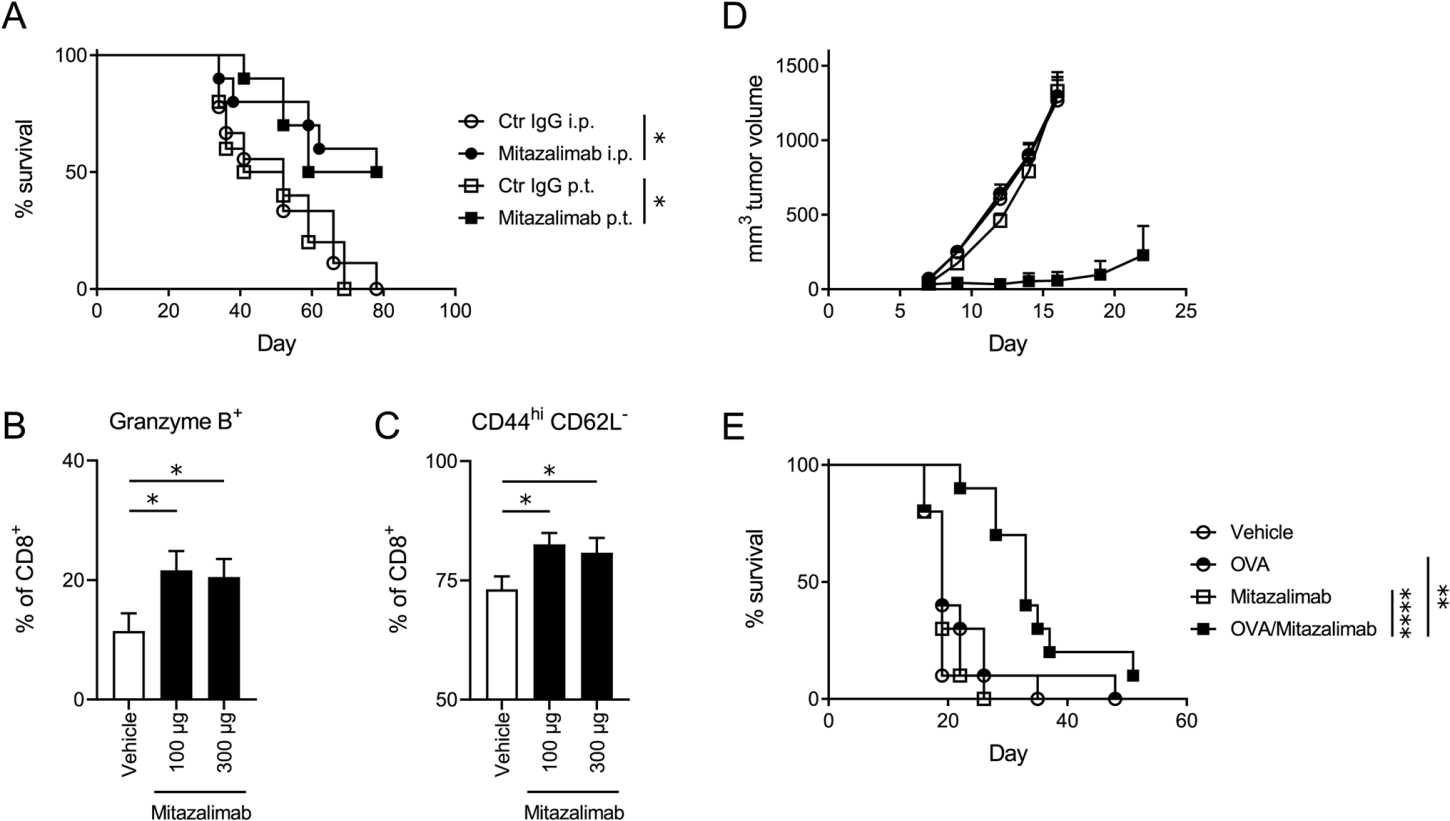
Therapeutic treatment with mitazalimab prolongs the survival of MB49 tumor-bearing mice and, combined with OVA peptide, also prolongs the survival of E.G7-OVA tumor-bearing mice**—**Mice (hCD40tg) were inoculated with 0.25 × 10^6^ MB49 cells s.c. and administered 100 µg mitazalimab or Ctr IgG p.t. or i.p. on day 7, 10 and 13 post-inoculation. **a** Frequency of survival throughout the study. Mice were sacrificed once their tumor volumes approached the ethical tumor volume limit of 2 cm^3^. Alternatively, the MB49 tumor-bearing mice were administered 100 or 300 µg mitazalimab i.p. on day 7, 10 and 13 post-inoculation and 24 h after the final dose, tumors were collected for flow cytometry, **b** frequency of granzyme B^+^ CD8^+^ T cells in the tumor, **c** frequency of CD44^hi^ CD62L^−^ CD8^+^ T cells in the tumor. Mice were inoculated with 1 × 10^6^ E.G7-OVA cells s.c. on one flank and administered 100 µg mitazalimab and/or 10 µg OVA peptide (SIINFEKL) s.c. on the other flank. The treatments were administered on the day of tumor inoculation and once more, 7 days later, **d** E.G7-OVA tumor growth throughout the study, **e** frequency of survival throughout the study. *n* = 9–10 per group in **a**; *n* = 8 per group in **b**–**c**; *n* = 10 per group in **d**–**e**. Statistical significance was analyzed by the log-rank test in **a** and **e**, and by Mann–Whitney *U* test in **b** and **c**. All error bars indicate ± SEM

Anti-tumor efficacy by therapeutic treatment with mitazalimab can be achieved in mice bearing syngeneic tumors. In the MB49 bladder carcinoma model, mitazalimab administered as single agent therapy resulted in a significantly prolonged survival compared to control IgG-treated mice, when given either p.t. or i.p. (Fig. 6a). Although mitazalimab administered i.p. did not result in increased frequency of total tumor-infiltrating CD8^+^ T cells (Suppl. Figure 3a), an increased activation of these cells was observed as demonstrated by the increased frequency of granzyme B^+^ CD8^+^ T cells (Fig. 6b), as well as CD44^hi^ CD62L^−^ effector memory CD8^+^ T cells in the tumor tissue (Fig. 6c).

To demonstrate that mitazalimab can improve cancer vaccine in a more clinically relevant setting, mice were given s.c. injections of mitazalimab and/or OVA peptide, rather than OVA protein, as therapeutic treatment. Thus, treatments were administered after E.G7-OVA tumor inoculation, on the same day, and once more 7 days later. Similar to previous observations, s.c. administration of mitazalimab and OVA peptide resulted in a significant expansion of OVA-specific CD8^+^ T cells 24 h after the second immunization (data not shown). Such therapeutic treatment also significantly delayed tumor growth and prolonged survival compared to mice receiving mitazalimab or OVA peptide monotherapy (Fig. 6d, e). A similar result was achieved when treatment was initiated at day 3 post-inoculation and the second dose was given on day 10, when the E.G7-OVA tumors had grown to a volume of approx. 200–250 mm^3^ (Suppl. Figure 3b). Mitazalimab thus has the capacity to enhance anti-tumor efficacy of cancer vaccines both as prophylactic and therapeutic treatment.

## Discussion

To enable treatment of patients with low tumor T cell infiltration, there is need for therapies capable of inducing priming of T cells in order to expand and activate the tumor-specific T cell repertoire. Stimulation of CD40 on DC enhances their tumor antigen cross-presentation and provides activation signals that mediate effective T cell priming [11–13]. This process can be bolstered by increasing the availability of tumor antigen accessible for uptake by DC and include therapeutic cancer vaccines, chemotherapy and radiotherapy.

Cancer vaccines are means to provide cancer antigens [25]; however, the resulting T cell responses will not be sufficient to eradicate tumors without effective cross-presentation by DC [12]. CD40 stimulation on DC induces a non-redundant pathway, enabling DC to efficiently activate CD8^+^ T cells also in the absence of helper T cells [26]. Numerous preclinical studies support the critical role of CD40 activation in a vaccination setting; for example, co-administration of a CD40 agonist with an HPV16 E7-derived peptide resulted in prolonged survival of mice bearing HPV16 E7-transduced tumors [27, 28]. Similar effects of a CD40 agonist were more recently observed in mice vaccinated with DC loaded with tumor lysate [29], and such effects could be potentiated in combination with adjuvants such as poly I:C [30–32]. CD40 agonists have also shown potential to improve T cell responses with vaccines against HIV antigens [33], and various pathogens [34, 35].

Mitazalimab was capable of activating DC, based on cell surface levels of the co-stimulatory markers CD80 and CD86, and this activation peaked within 48 h. Although such observations have been demonstrated for the murine surrogate anti-CD40 antibody FGK45 [36], these results confirm that a similar DC activation kinetic profile can be achieved by mitazalimab as well. The activation kinetics induced by mitazalimab on B cells, however, differed from those of the DC as B cell activation peaked only hours after treatment and was only detectable on a fraction of the total population. This is likely related to the hCD40tg mouse strain used in these studies, as these mice display a lower expression of human CD40 on B cells, when compared to the expression of murine CD40 on B cells in wildtype mice [20]. Still, it can be speculated that mitazalimab treatment may result in upregulation of not only CD80 and CD86, but also hCD40, as the B cells are activated. This could explain the effects of mitazalimab on B cells in hCD40tg mice, despite their low baseline expression of hCD40.

In clinical studies, common biomarkers for the activity of CD40 agonists include reduced numbers of circulating B cells and increased cell surface levels of CD80, CD86 or CD54 on remaining B cells [21–24], which correlates with the observations demonstrated in hCD40tg mice. A recent study suggested that the reduction in numbers of circulating B cells mediated by CD40 agonists could be the result of migration of these cells to the spleen [37]. Although we have not been able to demonstrate that mitazalimab treatment increases the frequency of splenic B cells in our model, it is possible that there is an equal numerary increase in splenic B cells and other immune cells as repeated mitazalimab treatment tends to increase the total cell number in the spleen. Treatment with mitazalimab also induced the secretion of proinflammatory chemokines and cytokines in the blood, and among those detected in the hCD40tg mice, IL-6, IP-10, MCP-1, MIP-1α, and TNF-α have also been detected in patients dosed with mitazalimab, with good tolerability profile [18, 19]. These data and the effects on circulating B cells thus confirm the immune-activating capacity of mitazalimab and support the hCD40tg mouse strain as a translationally relevant model.

A limitation with the current study is the lack of detailed analyses of B cell responses, as B cells are emerging as potentially important regulators of cancer immunity [38]. For example, a recent study highlighted the role of B cells and tertiary lymphoid structures in regulating responses to CPI [39]. Still, the role of tumor-infiltrating B cells in tumor progression has remained a controversial issue, as data indicate both positive and negative effects of these cells [38, 40].

In the case of CD40 agonists, the optimal dose regimen may be linked to the kinetics of the activation status of the responding DC, rather than the kinetics of the drug and receptor saturation over time. However, it is not clear if continuous activation of DC is optimal for achieving a potent anti-tumor response. In the present study, different mitazalimab dose regimens were evaluated in terms of both T cell priming and anti-tumor efficacy. Based on the results from the DC activation kinetics, mitazalimab was administered every 2–3 days, which is expected to result in continuously activated DC, or every 7 or 14 days, which should allow DC activation to return to baseline before the DC are again fully activated. Although these dose regimens resulted in comparable T cell priming in the OVA vaccination model and were capable of delaying E.G7-OVA tumor growth combined with OVA protein, only the dose regimen where mitazalimab was administered every 7 days significantly prolonged the survival of the mice. While this could indicate that too frequent exposure to mitazalimab results in depletion of DC via antibody-dependent cellular cytotoxicity and thus a reduced anti-tumor efficacy, there are no reports of preclinical or clinical studies that suggest that anti-CD40 antibodies mediate depletion of DC, despite that most CD40 agonists used in preclinical models engage Fcγ receptors. As our data instead show an initial accumulation of splenic DC in the spleen and activation of these cells in mice receiving mitazalimab every 2–3 days, a potential explanation for this observation could be that the frequent exposure to mitazalimab results in exhaustion of DC, or potentially development of neutralizing anti-mitazalimab antibodies, thus rendering these cells less capable of efficient cross-presentation. Although previous in vivo studies have demonstrated that CD40 agonist antibody treatment may result in depletion of CD8^+^ T cells in tumor-bearing mice, these effects could be circumvented by co-administration with a cancer vaccine [41]. Data on the kinetics of CD40-mediated DC activation in human subjects is lacking, and it is difficult to translate these findings to a clinical setting. In a clinical study with the CD40 agonist selicrelumab, the lack of clinical response observed after weekly dosing was explained by chronic activation of the APC, leading to hyperstimulation of the immune system and T cell exhaustion by too frequent dosing [22]. Subsequent clinical studies have used dose regimens where CD40 agonists have been administered every second week, or with longer dosing intervals [16].

Mitazalimab combined with OVA immunization, both as prophylactic and therapeutic treatment, resulted in anti-tumor efficacy of E.G7-OVA. In this model, mitazalimab single agent therapy did not result in anti-tumor efficacy. This is likely due to multiple factors, including dose regimen, administration route and the status of the tumor microenvironment. When administered as monotherapy, either systemically or locally to MB49 tumor-bearing mice on days 7, 10 and 13, instead of weekly on two occasions as was done in the therapeutic vaccination experiments, mitazalimab significantly prolonged the survival of the mice. It should be noted that all studies presented herein use female mice and it cannot be excluded that tumor growth may differ between female and male mice. This is particularly relevant for the MB49 model, which expresses the male HY antigen, thus rendering the MB49 cells more immunogenic in female mice [42].

Treatment of MB49 tumors with mitazalimab also resulted in activation of intratumoral T cells, based on granzyme B expression, as well as effector memory phenotype which was also promoted on splenic CD4^+^ and CD8^+^ T cells. These observations are in line with earlier work on the murine anti-CD40 antibody FGK45, for which similar T cell activation profiles have been observed in both spleen and tumor [43, 44]. Although in the context of this manuscript we have not evaluated the impact of mitazalimab on T regulatory cells, treatment with FGK45 has been shown to attenuate the suppressive phenotype of intratumoral T regulatory cells [44].

The treatment of E.G7-OVA tumor-bearing mice resulted in an immunological memory specific for OVA. This suggests that the immune response is focused primarily to this specific tumor antigen, provided here as a model cancer vaccine, and that epitope spreading to additional tumor-specific antigens is not generated. However, such observations can be expected when vaccinating with a strong T cell antigen such as OVA, in a rapidly growing tumor model. This also underscores the need to combine CD40 agonist treatment with multiple different tumor antigen peptides, preferably tumor neoantigens, for a potent immune response to be generated against the tumor. Still, additional mechanisms may dampen the T cell responses, and it may be necessary to combine vaccination strategies with therapies that aim to overcome such mechanisms, such as PD-(L)1-targeting therapies. Combination with anti-PD-(L)1 is further motivated by the observation that CD40 agonist treatment is accompanied by an IFN-γ-mediated upregulation of PD-L1 on monocytes and macrophages [43, 45]. Mitazalimab also resulted in upregulation of PD-L1 on DC with similar kinetics as CD80 and CD86, and earlier studies on mitazalimab support combination therapy with anti-PD-1 antibody [18].

In summary, the results presented herein demonstrate that mitazalimab potently activates APC such as DC in vivo, which subsequently results in improved activation and expansion of antigen-specific T cells. The data also support the notion that mitazalimab holds great potential to improve the anti-tumor efficacy of cancer vaccines.

## Supplementary Information

Below is the link to the electronic supplementary material.

**Supplementary Figure 1**. Mice were treated according to the experimental set-up in Fig. 3A. Graph shows frequency of splenic DC (CD11c^+^ MHCII^+^). *n* = 3–4 per group. Statistical significance was analyzed by ordinary two-way ANOVA and Šídák’s multiple comparisons test. All error bars indicate ± SEM. **Supplementary Figure 2**. Mice were treated according to the experimental set-up in Fig. 4A. **A**, Frequency of ICOS^+^ splenic CD4^+^ T cells. **B**, Frequency of CD44^hi^ CD62L- splenic CD4^+^ T cells. **C**, Frequency of total splenic CD4^+^ T cells. **D**, Frequency of total splenic CD8^+^ T cells. *n* = 3–4 per group. Statistical significance was analyzed by Mann–Whitney *U* test. All error bars indicate ± SEM. **Supplementary Figure 3**. Mice were inoculated with 0.25 × 10^6^ MB49 cells s.c. and administered 100 or 300 µg mitazalimab i.p. on day 7, 10 and 13 post-inoculation. Twenty-four hrs after the final dose, tumors were collected for flow cytometry. **A**, Frequency of total CD3^+^ CD8^+^ T cells in the tumor. Mice were inoculated with 1 × 10^6^ E.G7-OVA cells s.c. on one flank and administered 100 µg mitazalimab and/or 10 µg OVA peptide (SIINFEKL) s.c. on the other flank. The treatments were administered on day 3 post-inoculation and once more, 7 days later. **B**, E.G7-OVA tumor growth throughout the study. *n* = 8 per group in A; *n* = 10 per group in B. Statistical significance was analyzed by Mann–Whitney *U* test in A. All error bars indicate ± SEM. (PDF 188 kb)
