# Supplementary figures and images for: The human anti-CD40 agonist antibody mitazalimab (ADC-1013; JNJ-64457107) activates antigen-presenting cells, improves expansion of antigen-specific T cells, and enhances anti-tumor efficacy of a model cancer vaccine in vivo
Source: Cancer Immunol Immunother. 2021 May 5;70(12):3629–42. doi: 10.1007/s00262-021-02932-5 (PMC8571159; doi:10.1007/s00262-021-02932-5)

Supplementary Figure 1

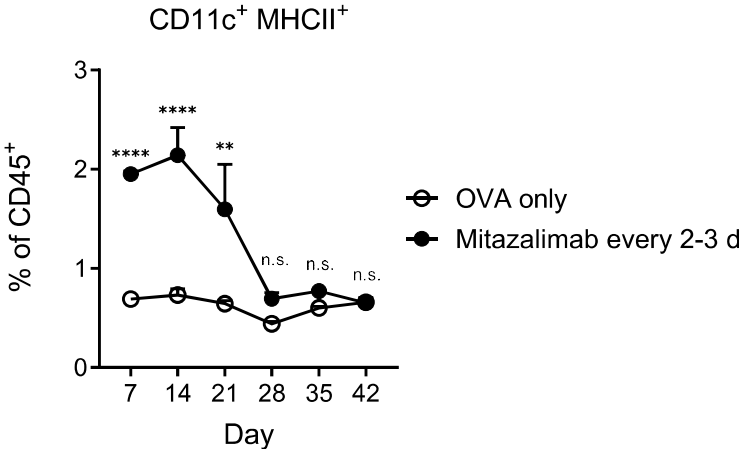

# Supplementary Figure 2

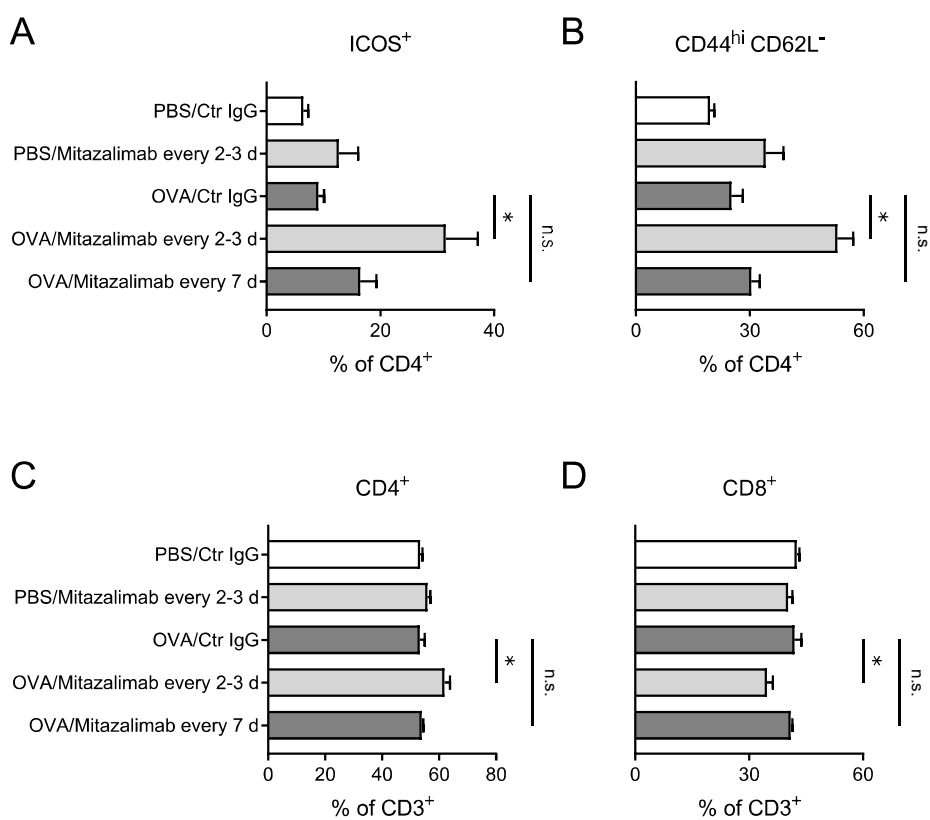

Supplementary Figure 3

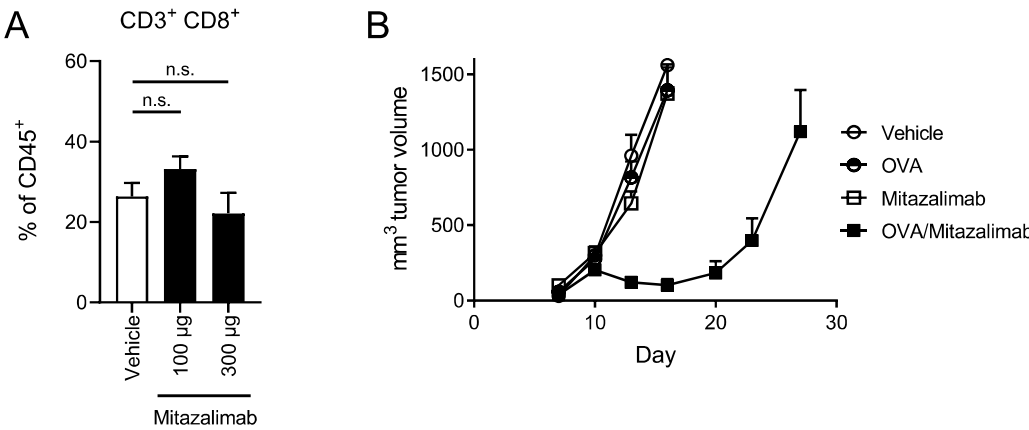

Supplement: Supplementary file 1 — Supplementary Figure 1. Mice were treated according to the experimental set-up in Fig. 3A. Graph shows frequency of splenic DC (CD11c+ MHCII+). n = 3–4 per group. Statistical significance was analyzed by ordinary two-way ANOVA and Šídák’s multiple comparisons test. All error bars indicate ± SEM. Supplementary Figure 2. Mice were treated according to the experimental set-up in Fig. 4A. A, Frequency of ICOS+ splenic CD4+ T cells. B, Frequency of CD44hi CD62L- splenic CD4+ T cells. C, Frequency of total splenic CD4+ T cells. D, Frequency of total splenic CD8+ T cells. n = 3–4 per group. Statistical significance was analyzed by Mann–Whitney U test. All error bars indicate ± SEM. Supplementary Figure 3. Mice were inoculated with 0.25 × 10^6^ MB49 cells s.c. and administered 100 or 300 µg mitazalimab i.p. on day 7, 10 and 13 post-inoculation. Twenty-four hrs after the final dose, tumors were collected for flow cytometry. A, Frequency of total CD3+ CD8+ T cells in the tumor. Mice were inoculated with 1 × 10^6^ E.G7-OVA cells s.c. on one flank and administered 100 µg mitazalimab and/or 10 µg OVA peptide (SIINFEKL) s.c. on the other flank. The treatments were administered on day 3 post-inoculation and once more, 7 days later. B, E.G7-OVA tumor growth throughout the study. n = 8 per group in A; n = 10 per group in B. Statistical significance was analyzed by Mann–Whitney U test in A. All error bars indicate ± SEM. (PDF 188 kb) [file 262_2021_2932_MOESM1_ESM.pdf]
